# A dual hypoxic marker technique for measuring oxygenation change within individual tumors.

**DOI:** 10.1038/bjc.1998.459

**Published:** 1998-07

**Authors:** R. V. Iyer, E. Kim, R. F. Schneider, J. D. Chapman

**Affiliations:** Department of Radiation Oncology, Fox Chase Cancer Center, Philadelphia, PA 19111, USA.

## Abstract

Rodent tumour models have been the 'workhorse' for tumour oxygenation research and for investigating radiobiological hypoxic fraction. Because of the intertumour heterogeneity of blood flow and related parameters, most studies have pooled information derived from several different tumours to establish the statistical significance of specific measurements. But it is the oxygenation status of and its modulation in individual tumours that has important prognostic significance. In that regard, the bioreducible hypoxic marker technique was tested for its potential to quantify oxygenation changes within individual tumours. Beta-D-iodinated azomycin galactoside (IAZG) and beta-D-iodinated azomycin xylopyranoside (IAZXP) were each radiolabelled with Iodine-125 and iodine-131 for measurements of animal tumour oxygenation. The tumour-blood (T/B) ratio of marker radioactivity in mice after the renal excretion of unbound marker (at 3 h and longer times) had been shown to be proportional to radiobiological hypoxic fraction. When markers labelled with both radioisotopes were administered simultaneously to EMT-6 tumour-bearing scid mice, T/B ratios were found to vary by up to 300% between different tumours, with an average intratumour variation of only approximately 4%. When the markers were administered 2.5-3.0 h apart, changes in T/B ratios of 8-25% were observed in 10 out of 28 (36%) tumours. Changes to both higher and lower hypoxic fraction were observed, suggestive of acute or cycling hypoxia. When 0.8 mg g(-1) nicotinamide plus carbogen was administered to increase tumour oxygenation, reductions in T/B ratios (mean deltaT/B approximately 38%) were observed in all tumours. Similar results were obtained with Dunning rat prostate carcinomas growing in Fischer x Copenhagen rats whose T/B ratios of IAZG and radiobiological hypoxic fractions are significantly lower. These studies suggest that fluctuating hypoxia can account for at least 25% of the total hypoxic fraction in some tumours and that correlations between bioreducible marker avidity and related tumour properties will be optimal when the independent assays are performed over the same time period. This dual hypoxic marker technique should prove useful for investigating both spontaneous and induced oxygenation changes within individual rodent tumours.


British Journal of Cancer (1998) 78(2), 163–169
© 1998 Cancer Research Campaign

### A dual hypoxic marker technique for measuring oxygenation change within individual tumors

**RV Iyer, E Kim, RF Schneider and JD Chapman**

Department of Radiation Oncology, Fox Chase Cancer Center, 7701 Burholme Avenue, Philadelphia, PA 19111, USA

**Summary** Rodent tumour models have been the 'workhorse' for tumour oxygenation research and for investigating radiobiological hypoxic fraction. Because of the intertumour heterogeneity of blood flow and related parameters, most studies have pooled information derived from several different tumours to establish the statistical significance of specific measurements. But it is the oxygenation status of and its modulation in individual tumours that has important prognostic significance. In that regard, the bioreducible hypoxic marker technique was tested for its potential to quantify oxygenation changes within individual tumours. β-D-Iodinated azomycin galactoside (IAZG) and β-D-iodinated azomycin xylopyranoside (IAZXP) were each radiolabelled with Iodine-125 and iodine-131 for measurements of animal tumour oxygenation. The tumour–blood (T/B) ratio of marker radioactivity in mice after the renal excretion of unbound marker (at 3 h and longer times) had been shown to be proportional to radiobiological hypoxic fraction. When markers labelled with both radioisotopes were administered simultaneously to EMT-6 tumour-bearing *scid* mice, T/B ratios were found to vary by up to 300% between different tumours, with an average intratumour variation of only ~4%. When the markers were administered 2.5–3.0 h apart, changes in T/B ratios of 8–25% were observed in 10 out of 28 (36%) tumours. Changes to both higher and lower hypoxic fraction were observed, suggestive of acute or cycling hypoxia. When 0.8 mg $g^{-1}$ nicotinamide plus carbogen was administered to increase tumour oxygenation, reductions in T/B ratios (mean ΔT/B ~38%) were observed in all tumours. Similar results were obtained with Dunning rat prostate carcinomas growing in Fischer X Copenhagen rats whose T/B ratios of IAZG and radiobiological hypoxic fractions are significantly lower. These studies suggest that fluctuating hypoxia can account for at least 25% of the total hypoxic fraction in some tumours and that correlations between bioreducible marker avidity and related tumour properties will be optimal when the independent assays are performed over the same time period. This dual hypoxic marker technique should prove useful for investigating both spontaneous and induced oxygenation changes within individual rodent tumours.

**Keywords:** hypoxic fraction; bioreducible marker; radioresistance; microenvironment; nicotinamide; carbogen

The oxygenation status of solid human tumours is one of several factors known to modulate their response to radiotherapy and ultimate tumour cure. Hypoxic cells are 2.5–3.0 times more resistant to killing by ionizing radiation (Gray et al, 1953; Chapman et al, 1974) and can be inaccessible for the delivery of and relatively resistant to some chemotherapy drugs (Tannock et al, 1981). Their selective survival, reoxygenation and eventual proliferation can produce local failure after treatment. Many factors associated with tumour cells and their interactions with host cells can modulate the oxygenation status of individual tumours as well as the number of clonogenic cells that are hypoxic and maximally radioresistant (Chapman, 1997).

Hypoxic fraction (HF) has traditionally been defined as a macroscopic tumour property, most commonly determined by the paired survival curve assay (Van Putten and Kallman, 1968; Moulder and Rockwell, 1984). This technique measures the clonogenicity of cells released from solid tumours that have been irradiated with graded doses in air-breathing and asphyxiated animals. In fact, this procedure is usually performed with five to ten tumours per treatment group to produce an average value of HF; it has proved to be useful for defining average HF as a function of tumour cell histology, implantation site and volume (Moulder and Rockwell, 1984). The distribution of HF values of individual tumours around their mean value can vary significantly and may be indicative of the intertumour heterogeneity of this specific property. Several different invasive and non-invasive assays of tumour oxygenation are under development for quantifying this tumour property before, during and after various therapies (Stone et al, 1993; Chapman, 1997). In that regard, bioreducible hypoxic markers have the potential for measuring the proportion of viable hypoxic cells within individual tumours (Chapman et al, 1981).

Chronic hypoxia in tumours results from the proliferation of their cells to volumes beyond those that can be adequately supplied with oxygen delivered through tumour vasculature (Chapman, 1997). Such tumour growth can lead to cords and necrosis, which have been observed by conventional histology (Thomlinson and Gray, 1958). More recently, intratumour pressure was postulated to produce transient vascular occlusions that would lead to local and acute areas of intermittent hypoxic cells (Brown, 1979). The presence of cycling hypoxia in rodent tumour models was elegantly demonstrated by a mismatch assay using two fluorescent and vascular-specific probes (Trotter et al, 1989*a* and *b*). This verification of acute or cycling hypoxia indicates that the HF of a specific tumour may vary significantly over time.

Two bioreducible hypoxic markers developed for the non-invasive detection of tumour hypoxia using nuclear medicine procedures (Schneider et al, 1997) were used to investigate spontaneous and induced oxygenation changes within individual rodent

*Received 7 October 1997*
*Revised 15 January 1998*
*Accepted 17 January 1998*

*Correspondence to*: JD Chapman tumours. Differences in tumour avidity for these markers labelled with different radioisotopes of iodine and administered before and after oxygen modulation procedures were suggestive of changes in tumour HF. This technique has produced minimal estimates of the contribution of acute hypoxia to the HF of some animal tumours and could find a useful role in other tumour oxygenation research.

### MATERIALS AND METHODS

### Hypoxic markers

The hypoxic markers used in this study were β-D-iodinated azomycin galactoside (IAZG) and β-D-iodinated azomycin xylopyranoside (IAZXP). They were synthesized locally and radiolabelled using procedures that have been reported previously (Schneider et al, 1997). The kinetics of their covalent linkage to molecules within tumour cells in vitro and mouse tumours in vivo have also been reported (Chapman et al, 1996; Schneider et al, 1997). Of the several iodinated azomycin nucleosides tested as hypoxic markers to date, IAZG and IAZXP have exhibited the optimal properties (Chapman et al, 1996). In the current study, IAZG and IAZXP were each radiolabelled with iodine-125 and iodine-131 to specific activities of 5–10 GBq $mmol^{-1}$. Each measurement of hypoxia in mouse and rat tumours was made with 50–80 kBq and 500–800 kBq, respectively, of radiolabelled markers at a maximum concentration within animal tissues of 0.5–0.7 μM.

### Animal tumour models

EMT-6 tumours (Rockwell et al, 1972) were grown in C.B17/Icr *scid* mice bred at the Fox Chase Cancer Center. EMT-6 cells growing in culture flasks were trypsinized, washed in sterile physiological saline [phosphate-buffered saline (PBS)], concentrated by centrifugation and resuspended at $2–5 \times 10^6$ cells $ml^{-1}$ in PBS. Aliquots (0.02 ml) of cell suspensions were injected subcutaneously on the upper backs of mice. This procedure produced solid tumours of 0.2–0.5 g at about 10 days after implantation (Chapman et al, 1996). Hypoxic marker measurements of tumour oxygenation were performed with tumours whose weights were usually between 0.2–0.8 g and whose HFs did not vary widely from a mean value of 35–40%.

The HFs of Dunning rat prostate carcinomas (R3327-AT and R3327-H) growing in Fischer × Copenhagen rats were also investigated. Host animals were bred at the Fox Chase Cancer Center. Tissue from a donor tumour was dissected into fragments of ~10 $mm^3$ in sterile PBS. Under mild anaesthesia with metophane, a skin area on the back of each rat was shaved, sterilized with 95% alcohol and incised to create a slit of ~1 cm length. One tumour fragment was inserted subcutaneously through the slit, which was then closed with a metal clip. Tumour growth was monitored twice weekly until tumours reached volumes of 5–10 $cm^3$ as determined from three mutually perpendicular measurements of tumour diameter made with callipers (Thorndyke et al, 1985). Previous studies (Thorndyke et al, 1985; Chapman et al, 1991; Moore et al, 1992, 1993) had shown R3327-AT tumours to exhibit both inter- and intratumour heterogeneity of blood flow and perfusion with an average radiobiological HF of ~15%. The well-differentiated R3327-H tumour showed no evidence of significant HF by radiobiological and hypoxic marker assays (Thorndyke et al, 1985). All animal tumour procedures were reviewed and approved by our local Animal Care and Use Committee.

### Measurement of tumour avidity for hypoxic marker

The partition coefficients (octanol–water) of IAZG and IAZXP are close to 1.0, which facilitated their rapid and relatively equal distribution to all animal tissues (Schneider et al, 1997). Excretion of radiolabelled markers from these mice and rats was biphasic. Over 95% of the marker was excreted renally with blood half-lives of about ~0.5 and ~1 h for mice and rats, respectively, after which time a much slower phase of hepatobiliary excretion of metabolized drug was observed. Previous studies had shown that maximal tumour–blood (T/B) levels of IAZG after its renal excretion phase (at 3 h and longer) were indicative of hypoxia in EMT-6 tumours as measured by radioresistance (Chapman et al, 1996). In our current study, reductions in T/B ratios of a marker labelled with different isotopes were interpreted as being indicative of increases in tumour oxygenation, whereas increases in T/B ratios of a marker were indicative of changes to higher HF. To derive quantitative estimates of radiobiological HF from T/B ratios with a specific marker, a calibration curve must be experimentally generated for each tumour model.

Radioactivity in samples of blood (B), muscle (M) and tumour (T) was measured with a Packard Cobra II Gamma Spectrometer operating in a dual-channel mode. The upper energy channel was adjusted to measure radioactivity from iodine-131 only. The lower

**Figure 1** (**A**) The %ID $g^{*-1}$ of [$^{131}$I]IAZG (open symbols) administered at time = 0 and [$^{125}$I]IAZG (closed symbols) administered at time = 3 h in the blood (○, ●), muscle (□, ■) and tumour (△, ▲) tissues of EMT-6 tumour-bearing C.B17/Icr *scid* mice (*n* = 5). (**B**) The tumour/blood (triangles) and tumour/muscle (squares) ratios of specific tissue radioactivity (%ID $g^{*-1}$) of [$^{131}$I]IAZG (open symbols) and [$^{125}$I]IAZG (closed symbols) at various times after administration

© Cancer Research Campaign 1998

energy channel, which exclusively measured Iodine-125, was adjusted so that spilldown counts from iodine-131 were a small percentage of the total. Radioactivity from each isotope obtained as d.p.m. were back-corrected for decay, corrected for counting efficiency and converted to per cent injected dose per gram of tissue (%ID $g^{-1}$). As the body weights of the animals varied slightly, the tissue-specific activities for each mouse were adjusted to a standard mouse (20 g*) using the factor mouse weight/20. T/B and T/M ratios were calculated from such values of normalized tissue specific activity (%ID $g^{*-1}$).

### Tumour oxygen modulation

Markers labelled with iodine-125 and iodine-131 were administered simultaneously to some tumour-bearing animals and 2.5–3.0 h apart to others. Tumour oxygenation was modulated by administration of 0.8 mg $g^{-1}$ nicotinamide plus carbogen, which had been shown to significantly reduce the hypoxic fractions in some rodent tumours (Horsman et al, 1988, 1990; Chaplin et al, 1991). Nicotinamide was purchased from Sigma, prepared in sterile PBS at 160 mg $ml^{-1}$ and administered i.p. to a final concentration of 0.8 mg $g^{-1}$. Carbogen gas was purchased as a mixture from BOC gases and flowed at 3 l $min^{-1}$ through sealed cages. Tumour-bearing animals were placed in these cages and breathed carbogen at 1 atmospheric pressure throughout the second marker procedure.

### Statistical evaluations

T/B ratios of the markers administered at different times were subtracted to indicate absolute and percentage changes in HF. Significance of changes in mean value of T/B ratio between groups of animals was analysed using Student's *t*-test or the Mann–Whitney test. Variations in T/B ratios observed within groups of similarly treated animals are reported as standard errors of the mean (s.e.m.).

### RESULTS

The biodistribution and pharmacokinetics of single administrations of IAZG and IAZXP to EMT-6 tumour-bearing *scid* mice have been reported previously (Chapman et al, 1996; Schneider et al, 1997). Additional studies were performed to determine whether the pharmacokinetics of one marker dosage were altered by administration of the same marker at a different time. Figure 1A shows the %ID $g^{*-1}$ of [$^{131}$I]IAZG and [$^{125}$I]IAZG in blood, muscle and tumour tissues at various times after their administration 3 h apart to the same groups of EMT-6 tumour-bearing mice. The open symbols are specific radioactivities of the first administered marker (I-131), while the solid symbols are the specific radioactivities of the second marker (I-125) in the same tissues. Thirty minutes after the administration of each marker, all the sampled tissues contained marker at concentrations of 2.2–2.6%ID $g^{*-1}$. In this experiment, the rapid clearance rate of IAZG from blood and muscle was characterized by a half-life of ~35 min. Extrapolation of those data back to the time of injection yielded initial values of 4–5% ID $g^{*-1}$, a value close to that expected for the uniform distribution of marker to the soft tissues of a 20-g mouse. Selective uptake and retention in tumour tissue is apparent at 1 h and longer times after administration. After 3 h, the clearance of residual marker is by a much slower process related to hepatobiliary excretion. Figure 1B indicates that T/B and T/M ratios of marker reached maximum values after the rapid renal excretion phase of unbound marker. In this study, the maximal T/B and T/M ratios achieved with IAZG were ~11 and ~23 respectively. It is apparent that the pharmacokinetics of [$^{125}$I]-IAZG are similar to those of the [$^{131}$I]IAZG, which was administered 3 h earlier. When the tissue levels of the second administered marker were backshifted by 3 h in Figure 1A, the two data sets were found to superimpose. The symbols represent mean values from tissue sampled from five tumour-bearing animals at each timepoint and, in most cases, the s.e.m. was smaller than the size of the symbols used in both Figures 1A and B. These data give us confidence that this hypoxic marker can be administered to the same animals at these concentrations with little or no interference of one assay with the other.

**Table 1** The oxygenation status of EMT-6 tumours growing in C.B17/Icr *scid* mice ($n$ = 12) as measured by the maximal tumour–blood (T/B) ratios of the hypoxic markers [$^{125}$I]IAZXP and [$^{131}$I]IAZXP when administered simultaneously

| Weight (g) | T/B ($^{125}$I) | T/B ($^{131}$I) | ΔT/B | % Change |
|---|---|---|---|---|
| 0.17 | 12.5 | 13.3 | +0.8 | 6.4 |
| 0.18 | 5.5 | 5.3 | −0.2 | 3.6 |
| 0.20 | 11.9 | 11.9 | 0 | 0 |
| 0.29 | 10.9 | 12.0 | +1.1 | 10.1 |
| 0.30 | 9.9 | 10.4 | +0.5 | 5.0 |
| 0.35 | 13.2 | 13.1 | −0.1 | 0.8 |
| 0.36 | 12.7 | 12.8 | +0.1 | 0.8 |
| 0.38 | 13.9 | 14.1 | +0.2 | 1.4 |
| 0.44 | 10.4 | 10.3 | −0.1 | 1.0 |
| 0.47 | 10.6 | 9.8 | −0.8 | 7.5 |
| 0.51 | 11.3 | 10.9 | −0.4 | 3.5 |
| 0.56 | 11.1 | 10.2 | −0.9 | 8.1 |
| 0.35 ± 0.04[a] | 11.2 ± 0.6 | 11.2 ± 0.7 | 0.4 | 4.0 |

[a]Mean ± s.e.m.

**Table 2** Maximal tumour–blood (T/B) ratios of [$^{125}$I]IAZXP and [$^{131}$I]IAZXP when administered 2.5 h apart for EMT-6 tumours of different size ($n$ = 18). The asterisks indicate % change in the T/B ratios greater than 8

| Weight (g) | T/B ($^{125}$I) | T/B ($^{131}$I) | ΔT/B | % Change |
|---|---|---|---|---|
| 0.21 | 11.3 | 10.8 | −0.5 | 4.4 |
| 0.22 | 12.0 | 11.5 | −0.5 | 4.2 |
| 0.25 | 10.2 | 11.8 | +1.6 | 15.7* |
| 0.25 | 11.5 | 11.7 | +0.2 | 1.7 |
| 0.26 | 11.5 | 11.3 | −0.2 | 1.7 |
| 0.38 | 8.3 | 7.3 | −1.0 | 12.0* |
| 0.43 | 9.8 | 10.0 | +0.2 | 2.0 |
| 0.44 | 10.3 | 9.8 | −0.5 | 4.9 |
| 0.45 | 11.6 | 11.2 | −0.4 | 3.4 |
| 0.68 | 9.8 | 9.3 | −0.5 | 5.1 |
| 0.48 | 13.6 | 12.6 | −1.0 | 7.4 |
| 0.51 | 11.9 | 11.2 | −0.7 | 5.9 |
| 0.53 | 12.7 | 11.7 | −1.0 | 7.9 |
| 0.56 | 11.2 | 9.6 | −1.6 | 14.3* |
| 0.60 | 11.0 | 8.8 | −2.2 | 20.0* |
| 0.65 | 10.8 | 11.8 | +1.0 | 9.3* |
| 0.68 | 9.8 | 9.3 | −0.5 | 5.1 |
| 0.68 | 12.3 | 12.3 | 0 | 0 |
| 0.76 | 10.3 | 8.7 | −1.5 | 14.6* |
| 0.46 ± 0.04[a] | 11.1 ± 0.29 | 10.6 ± 0.34 | 0.8 | 7.5 |

[a]Mean ± s.e.m.

© Cancer Research Campaign 1998

**Table 3** Maximal tumour/blood (T/B) ratios of [$^{125}$I]IAZG when administered 3 h before [$^{131}$I]IAZG and tumour oxygen modulation by 0.8 mg g$^{-1}$ nicotinamide plus carbogen

| Weight (g) | T/B pre | T/B post | ΔT/B | % Change |
|---|---|---|---|---|
| *Control* | | | | |
| 0.27 | 14.5 | 14.7 | +0.2 | 1.4 |
| 0.28 | 14.1 | 14.4 | +0.3 | 2.1 |
| 0.31 | 12.0 | 14.6 | +2.6 | 21.7 * |
| 0.35 | 11.7 | 10.8 | −0.9 | 7.7 |
| 0.41 | 11.3 | 12.1 | +0.8 | 7.1 |
| 0.41 | 13.5 | 12.6 | −0.9 | 6.7 |
| 0.43 | 13.3 | 14.6 | +1.3 | 9.8 * |
| 0.43 | 14.2 | 14.1 | −0.1 | 0.7 |
| 0.43 | 13.3 | 12.2 | −1.1 | 8.3 * |
| 0.44 | 13.1 | 10.4 | −2.7 | 20.6 * |
| 0.38 ± 0.02[a] | 13.1 ± 0.35 | 13.1 ± 0.52 | 1.1 | 8.6 |
| *Modulated* | | | | |
| 0.26 | 15.3 | 9.9 | −5.4 | 35.3 |
| 0.30 | 16.2 | 10.1 | −6.1 | 37.7 |
| 0.33 | 15.8 | 7.8 | −8.0 | 50.6 |
| 0.33 | 18.8 | 8.9 | −9.9 | 52.7 |
| 0.34 | 13.3 | 8.2 | −5.1 | 38.3 |
| 0.35 | 16.9 | 10.0 | −6.9 | 40.8 |
| 0.43 | 14.7 | 10.2 | −4.5 | 30.6 |
| 0.45 | 17.4 | 9.9 | −7.5 | 43.1 |
| 0.46 | 15.1 | 10.7 | −4.4 | 29.1 |
| 0.50 | 16.2 | 13.5 | −2.7 | 16.7 |
| 0.38 ± 0.03[a] | 16.0 ± 0.48 | 9.9 ± 0.49 | 6.1 | 37.5 |

[a]Mean ± s.e.m. Control group ($n$ = 10). Modulated group ($n$ = 10). The asterisks indicate % change in T/B ratios greater than 8 in control animals.

**Table 4** Changes in T/B ratios of radiolabelled IAZG in R3327-AT tumour-bearing rats before and after modulation with 0.8 mg g$^{-1}$ nicotinamide and carbogen gas

| Weight (g) | T/B Pre | T/B Post | ΔT/B | % Change |
|---|---|---|---|---|
| *Control* | | | | |
| 5.6 | 2.1 | 2.3 | +0.2 | 9.5 |
| 7.4 | 2.8 | 3.1 | +0.3 | 10.7 |
| 7.7 | 2.2 | 2.4 | +0.2 | 9.1 |
| 9.6 | 2.6 | 2.8 | +0.2 | 7.7 |
| 10.2 | 3.6 | 3.8 | +0.2 | 5.6 |
| 10.8 | 3.0 | 3.4 | +0.4 | 13.3 |
| 10.9 | 2.3 | 2.2 | −0.1 | 4.3 |
| 18.7 | 3.7 | 4.2 | +0.5 | 13.5 |
| 10.1 ± 1.39[a] | 2.8 ± 0.22 | 3.0 ± 0.26 | 0.26 | 9.2 |
| *Modulated* | | | | |
| 8.6 | 2.9 | 1.4 | −1.5 | 52 |
| 8.9 | 2.1 | 1.1 | −1.0 | 48 |
| 10.5 | 2.4 | 1.6 | −0.8 | 33 |
| 11.4 | 2.2 | 1.6 | −0.6 | 27 |
| 11.5 | 3.3 | 0.7 | −2.6 | 79 |
| 12.1 | 3.3 | 1.6 | −1.7 | 52 |
| 13.7 | 3.4 | 1.8 | −1.6 | 47 |
| 13.9 | 2.7 | 2.2 | −0.5 | 19 |
| 11.3 ± 0.69 | 2.8 ± 0.18 | 1.5 ± 0.16 | 1.3 | 45 |

[a]Mean ± s.e.m.

Additional preliminary studies were performed to evaluate the precision of our dual-channel γ-scintigraphic procedure. IAZXP labelled with each iodine isotope was administered i.p. at the same time to EMT-6 tumour-bearing mice. After 5 h, the animals were sacrificed and samples from tumour, muscle and blood were obtained, weighed and analysed for [$^{131}$I]IAZXP and [$^{125}$I]IAZXP. Table 1 shows the tumour weights, the T/B ratios of % ID g*$^{-1}$ for each marker, the absolute difference in T/B ratio (ΔT/B) and the percentage difference in T/B ratios measured by the two markers. Twelve animals with tumours of 0.17–0.56 g were used in this investigation. T/B ratios of hypoxic marker varied from 5.3 to 14.1. In spite of this large intertumour variation in apparent HF, the mean value of T/B ratio measured in all the tumours with each marker was identical ($P$ = 0.923). Each marker clearly delineated the tumours with the lowest and the highest T/B ratios. When changes in marker ratios for individual tumours were analysed, they were found to distribute almost equally to slightly higher and lower values. The average percentage difference in T/B ratios in individual tumours measured by the two markers was 4% and less than 10% for 11 of the 12 tumours. These data clearly demonstrate that the experimental error associated with the measurement of HF in individual tumours with two different bioreducible markers is very small relative to their intertumour variation of HF. When T/M ratios were analysed in all the studies, the results followed a pattern similar to that seen for the T/B ratios, although the actual numbers were proportionally higher.

The dual hypoxic marker procedure was used to determine whether or not significant changes in individual tumour HF occurred over relatively short times. Times of 2.5–3.0 h were initially investigated, as the data in Figure 1 suggested that little or no interaction occurred between markers at these times. Tumour-bearing *scid* mice were administered 50 kBq of [$^{125}$I]IAZXP 2.5 h before the administration of 50 kBq of [$^{131}$I]IAZXP. Three hours after the second marker administration the animals were sacrificed. Blood, muscle and tumour tissues were obtained, weighed and analysed for specific activities of each marker. Table 2 shows the tumour weights and values of T/B ratios of %ID g*$^{-1}$ from two experiments performed on different days with tumours of different volumes. The mean values of T/B ratios of [$^{125}$I]IAZXP and [$^{131}$I]IAZXP were similar and numerically close to those reported in Table 1. In 8 of 18 tumours investigated, the difference in T/B ratios of marker was 5% or less, suggestive of little or no change in their HF. We have defined percentage changes of T/B ratios of markers in individual tumours of 8% and greater (at least two times the average change reported in Table 1) to be indicative of significant change in tumour HF. By this criterion, 6 of the 18 tumours reported in Table 2 and four of the ten control tumours (similarly treated) in Table 3 had different HF at these different times. These tumours are indicated by asterisks (*) in the tables. It is reasonable to suggest that these changes result from cycling hypoxia mechanisms, as almost equal changes to higher and lower HF were observed.

The dual hypoxic marker technique was also used to investigate induced oxygenation change within individual tumours in animals treated with 0.8 mg g$^{-1}$ nicotinamide plus carbogen. Twenty *scid* mice with EMT-6 tumours were randomized to two treatment groups of ten animals each. For this study, [$^{125}$I]IAZG and [$^{131}$I]IAZG were used because of availability. This hypoxic marker produces slightly higher maximum values of T/B ratios than does IAZXP (Chapman et al, 1996). Control animals were administered 50 kBq of [$^{125}$I]IAZG 3 h before the administration of 50 kBq of

 © Cancer Research Campaign 1998

**Table 5** Changes in T/B ratios of radiolabelled IAZG in R3327-H tumour-bearing rats before and after modulation with 0.8 mg g$^{-1}$ nicotinamide and carbogen

| Weight (g) | T/B Pre | T/B Post | ΔT/B | % Change |
|---|---|---|---|---|
| *Control* | | | | |
| 6.5 | 1.4 | 1.7 | +0.3 | 21 |
| 8 | 1 | 0.9 | −0.1 | 10 |
| 8.7 | 1 | 0.7 | −0.3 | 30 |
| 8.9 | 0.8 | 0.8 | 0 | 0 |
| 8.9 | 0.9 | 0.8 | −0.1 | 11 |
| 9.9 | 0.9 | 0.7 | −0.2 | 22 |
| 11.6 | 0.7 | 0.6 | −0.1 | 14.3 |
| 17.9 | 1.2 | 1 | −0.2 | 16.7 |
| 10.1 ± 1.23[a] | 0.99 ± 0.08 | 0.90 ± 0.12 | 0.16 | 15.6 |
| *Modulated* | | | | |
| 6.8 | 1.1 | 1.2 | +0.1 | 9 |
| 7.4 | 0.8 | 0.9 | +0.1 | 12.5 |
| 7.7 | 0.8 | 1.1 | +0.3 | 37.5 |
| 9.7 | 1.6 | 1 | −0.6 | 37.5 |
| 10.7 | 1.3 | 0.8 | −0.5 | 38.5 |
| 13.9 | 1 | 1 | 0 | 0 |
| 14.5 | 1.1 | 1.4 | +0.3 | 27 |
| 16.8 | 1.4 | 1.2 | −0.2 | 14 |
| 10.9 ± 1.32 | 1.14 ± 0.10 | 1.08 ± 0.07 | 0.3 | 22 |

[a]Mean ± s.e.m.

[$^{131}$I]IAZG. Sterile PBS (0.1 ml) was administered i.p. with the second marker and, 3 h later, animals were sacrificed, tissues were sampled, weighed and specific activities of markers in tissues determined. The data for the control animals in Table 3 indicate that the mean values of tumour HF as measured by T/B ratios of IAZG were similar at the two times of observation ($P = 0.937$). For individual tumours, the percentage changes in T/B ratio were less than 8% in six of ten tumours and greater than 20% in two of ten tumours. In those tumours that showed an apparent change in HF (≥ 8% ΔT/B), two tumours were to a lower and two were to a higher HF. In the modulated group of animals, nicotinamide was administered in 0.1 ml of sterile PBS to a final concentration of 0.8 mg g$^{-1}$ with the second radiolabelled marker and the animals were placed in a sealed cage with carbogen gas flowing for 1 h. The data in Table 3 show a significant reduction (average change of ~38%) in T/B ratios of IAZG for this modulated group of tumours ($P < 0.0001$). In individual animals, the percentage reduction in apparent HF ranged from 17% to 53%. These data indicate that this dual-marker technique has the potential for measuring oxygenation change within individual rodent tumours.

The dual hypoxic marker technique was further investigated with Dunning R3327-AT and R3327-H tumours growing in Fischer × Copenhagen rats. The radiosensitivity, perfusion status and spontaneous and photodynamic therapy-induced hypoxia in these tumour models have been reported previously (Thorndyke et al, 1985; Chapman et al, 1991; Moore et al, 1992, 1993). The average radiobiological HF of R3327-AT tumours is ~15%, whereas the R3327-H tumour is well perfused with no measurable HF by the radiobiological or hypoxic marker techniques (Thorndyke et al, 1985). [$^{125}$I]IAZG was administered to tumour-bearing rats at the time of nicotinamide (0.8 mg g$^{-1}$) and carbogen administration (for 2 h), 5–6 h after an initial administration of [$^{131}$I]IAZG. Each marker was administered at 500–800 kBq and animals were sacrificed 14–16 h after the second marker administration, when T/B and T/M ratios were known to be at maximum plateau values. The clearance kinetics of these markers from Fischer × Copenhagen rats requires twice the time of the clearance observed with *scid* mice. Blood, muscle and tumour tissues were sampled, weighed and counted to determine the specific tissue radioactivity of each marker. Table 4 shows results for control ($n = 8$) and modulated rats ($n = 8$) in which R3327-AT tumours of 5–18 g were growing. The largest of these tumours weighed less than 5% of the rat weight of 400 g, on average. The volumes of these tumours were at least ten times larger than those of the EMT-6 tumours, but their percentages of body weights were similar. The absolute binding rate of IAZG to hypoxic R3327-AT cells in vitro is similar to the binding rate to hypoxic EMT-6 cells, when corrections are made for differences in their cell volumes (JD Chapman, unpublished data). In spite of this similarity in IAZG avidity for hypoxic tumour cells in vitro, the T/B ratios of IAZG in control R3327-AT tumours was ~3. This much lower uptake of IAZG into these tumours in vivo is mainly associated with their much smaller radiobiological HF. The administration of nicotinamide and carbogen to R3327-AT tumour-bearing rats produced a significant reduction ($P = 0.0001$) in mean T/B ratio of marker (average reduction of 45%). This proportional change is similar to that observed with the more hypoxic EMT-6 tumours and varies from 19% to 79% for individual tumours.

Similar investigations were performed with R3327-H tumour-bearing rats which exhibit little or no radiobiological hypoxia. Table 5 shows results from control ($n = 8$) and modulated ($n = 8$) groups of animals. Mean values of T/B ratios of IAZG before tumour modulation were close to 1.0, which indicates that these tumours contain few or no zones of hypoxic tumour cells. The mean values of T/B ratios measured with the two markers in the control group did not change significantly ($P = 0.234$). In both control and modulated tumours, T/B ratios of IAZG increased in five, decreased in nine and remained the same in two tumours. While the absolute differences in T/B ratios measured with the two markers are small, the same changes expressed as a percentage of the first measurement are relatively large because of the small denominator. There was no significant reduction ($P = 0.612$) in average T/B ratios of hypoxic marker induced by the nicotinamide and carbogen treatment. These data confirm that the intrinsic oxygenation status of R3327-H tumours is significantly better than that of R3327-AT tumours and that the reoxygenation effect induced by nicotinamide and carbogen in R3327-AT tumours was not measurable in R3327-H tumours.

### DISCUSSION

IAZG and IAZXP were chosen for this study because of their similar binding properties to viable hypoxic cells and their rapid plasma clearance half-lives (Chapman et al, 1996; Schneider et al, 1997). In addition, both could be readily radiolabelled with iodine-125 or iodine-131. Both markers rapidly distributed quite uniformly to all tissues after i.p. injection, achieved maximal tissue levels within 20–30 min and exhibited clearance half-lives of about 30 min in mice (Chapman et al, 1996). The initial clearance phase of unmetabolized drug was complete within 3 h. The binding of marker to hypoxic cells within animal tumours is dependent upon several pharmacological factors but occurs mainly over the first hour, when marker concentration is high, and becomes most evident after the clearance of unbound marker from

© Cancer Research Campaign 1998

the surrounding normal tissues. This bioreducible hypoxic marker procedure will have its highest sensitivity for measuring tumour HF after the initial clearance phase of unbound marker, when T/B and T/M ratios are maximum. It was at this time that T/B ratios of IAZG were shown to correlate with tumour radioresistance (Chapman et al, 1996).

For bioreducible hypoxic markers labelled with different radioisotopes to detect oxygenation change in individual tumours, several specific criteria had to be met. As this procedure is strongly dependent upon the biodistribution and pharmacokinetics of the marking agent, it was important to demonstrate that two dosages of the same marker labelled with different radioisotopes separated in time by a few hours did not interfere with their independent pharmacokinetics. The data presented in Figure 1 demonstrate that when [$^{125}$I]IAZG and [$^{131}$I]IAZG were administered 3 h apart to the same EMT-6 tumour-bearing animals, they showed little or no interaction. This result was expected as the maximum concentration of IAZG and IAZXP delivered to most mouse tissues was ~1 μM. If these same markers were administered at much higher concentrations (0.1 mM or greater) and/or at closer times, the probability of one dose affecting a second dose could be greater. The high specific activities of the radiolabelled markers used in this study produced a high sensitivity at these low concentrations.

The intrinsic precision of our dual-channel γ-counting procedure was determined with IAZXP labelled with the two radioisotopes of iodine administered simultaneously to EMT-6 tumour-bearing mice. The average percentage difference in T/B ratios of %ID $g^{*-1}$ of IAZXP was 4% (range from 0 to 10.1%). This represents the sum of errors associated with marker administration to animals, tissue necropsy, tissue weighing as well as the quantification of radioactivity decays from both iodine-125 and iodine-131 in the same samples. The data in Table 1 indicate a remarkably good consistency for predicting tumour HF from maximal T/B ratios with this hypoxic marker technique. Based upon these data, we suggested that changes in T/B ratios of hypoxic marker of 8% and greater (≥ two times average variation) in these tumours would indicate significant changes in tumour HF. According to this criterion, 10 of the 28 control and unmodulated tumours in Tables 2 and 3 demonstrated significant change in hypoxic fraction over 2.5–3.0 h. These are indicated by asterisks (*) in the tables. The average change in T/B ratio for these ten tumours was ~15% with four shifting to higher HF and six shifting to lower HF. This result suggests that acute or cycling hypoxia may account for at least 22% of the HF of some EMT-6 tumours. This appears to be a minor component of their total HF, but it would be incorrect to assume that the remaining HF results from only chronic mechanisms. On the other hand, bioreducible markers will be taken up maximally into viable tumour cells that reside at very low oxygen concentration for the duration of the marking procedure (1–2 h). Consequently, this technique is biased towards the labelling of chronically hypoxic cells and more than 80% of the marker grains in tumour autoradiographs from labelled misonidazole were associated with cells adjacent to necrosis (Chapman et al, 1982). While the dual-marker procedure provides additional evidence for the presence of acute or cycling hypoxia in some EMT-6 and R3327-AT tumours, it cannot determine its exact proportion. These data also showed that tumour HF measured by the bioreducible marker procedure did not change significantly over this time in two out of three of the tumours.

When $pO_2$ measurements were made in R3327-AT tumours with the Eppendorf $pO_2$ microelectrode, 24 h apart, a 60% difference in median $pO_2$ was observed over 24 h (Yeh et al, 1995). Median $pO_2$ was found to increase in nine and decrease in 6 of the 15 tumours used in that study. These electrode measurements could suggest that the contribution of acute and cycling hypoxia to HF of R3327-AT tumours is greater than that measured with these hypoxic markers, although microelectrode measurements were undoubtedly obtained from tumour tracks that crossed zones of both viable and dead tumour tissue. Bioreducible markers, on the other hand, estimate intracellular oxygen levels only in viable cells whose complement of reductive enzymes are intact. Consequently, hypoxic marker avidity to tumour tissue should more closely correlate with the HF of viable tumour cells. Indeed, when the ability of different hypoxic assays to predict for tumour radioresistance was investigated in some rodent tumours, tritiated misonidazole avidity to tumour best predicted for tumour radioresistance (Hu et al, 1995; Kavanagh et al, 1996). The proportion of tumour vessels that stained in murine SCCVII carcinomas by only one fluorescence probe showed a maximum of ~9% for larger tumours (Trotter et al, 1989*a*). Although these fluorescent probes elegantly demonstrated transient blood flow in rodent tumours, they yielded no information about chronic hypoxia. Consequently, the relative contribution of acute or cycling hypoxia to total tumour HF could not be estimated from those studies. The novel dual-hypoxic marker experiments described in this manuscript may indicate minimal estimates of acute relative to total tumour hypoxia.

The sensitivity of this dual-hypoxic marker technique for measuring oxygenation change induced in individual tumours was investigated using nicotinamide and carbogen. The concomitant administration of these agents to tumour-bearing animals has consistently shown significant reductions in tumour HF (Horsman et al, 1988, Chaplin et al, 1991). The HFs of EMT-6 and R3327-AT tumours estimated from T/B ratios of IAZG were consistently and significantly reduced by the administration of nicotinamide and carbogen. Although the radiobiological HF and T/B ratios of hypoxic marker were different for the two tumour models, the administration of nicotinamide and carbogen gas to tumour-bearing animals produced an average decrease in T/B hypoxic marker ratios of 38–45%. These reductions in tumour avidity for hypoxic marker strongly indicate an increased tumour oxygenation, consistent with radiobiological estimates of changes in tumour HF observed in other tumour models (Horsman et al, 1990). It should be noted that the effect of nicotinamide and carbogen on tumours in different animals was quite variable. Tumour HF appears to be altered by as little as 20% in some tumours and by as much as 80% in others. These data suggest that the dual-hypoxic marker procedure has the potential for quantifying induced as well as spontaneous changes in oxygenation status of individual rodent tumours. Unfortunately, the validation of such changes of individual tumour HF by specific pharmacological procedures will require independent assays performed on the same tumours. In addition, some tumour-modulating procedures are known to affect hypoxic marker pharmacokinetics and thus make comparisons of T/B ratios less informative. But the studies described in this manuscript clearly show that when different assays of tumour oxygenation are to be correlated with each other and with radiobiological HF, the assays should be performed at the same time. This poses a severe demand for correlations between different tumour oxygen assays and could prove to be impossible for some.

© Cancer Research Campaign 1998

The T/B ratio of IAZG for R3327-H rat prostate carcinomas was ~1.0 for most tumours. This result is indicative of good tumour oxygenation and consistent with our previous studies that showed no significant hypoxic fraction by radiobiological or hypoxic marker procedures (Thorndyke et al, 1985). The administration of nicotinamide and carbogen to R3327-H tumour-bearing animals did not significantly change the average T/B ratios of hypoxic marker. For some R3327-H tumours that exhibited T/B hypoxic marker ratios greater than 1.3, histological examination of resected tumour tissue at autopsy showed zones of only moderate differentiation. This hormone-responsive, well-differentiated rat prostate carcinoma usually has a latent period after implantation of 3–4 months and a growth doubling time of 12–20 days. The R3327-AT tumour is an anaplastic variant outgrown from this well-differentiated tumour. From these studies, we have come to learn that R3327-H tumours, which develop early after implantation, exhibit faster doubling times or exhibit T/B hypoxic marker ratios of greater than 1.5, are likely to contain zones of less differentiated and even anaplastic tumour, some of which can develop zones of hypoxia.

In summary, the data presented in this manuscript indicate that bioreducible hypoxic markers labelled with two radioisotopes (or other distinguishable signals) can be administered at different times to the same tumour-bearing animals for detection of changes in tumour oxygenation. T/B or T/M ratios are proportional to tumour radiobiological HF and changes in marker avidity ratios are consistent with changes in average tumour oxygenation. Of the various techniques described to date for monitoring tumour oxygenation, the dual-hypoxic marker procedure may have the greatest use for measuring average oxygenation change in individual rodent tumours. This technique has now identified intertumour heterogeneity for both spontaneous and induced oxygenation changes in experimental tumours. Our preliminary experiments suggest that acute or cycling hypoxia in EMT-6 mouse and R3327-AT rat tumours can account for, at least, up to 22% of total tumour HF in some tumours. This result requires validation by independent assays of tumour hypoxia, but suggests that correlations between various tumour oxygenation assays and tumour radioresistance should be performed simultaneously so that differences associated with changes in tumour HF will be minimized.

### ACKNOWLEDGEMENTS

Financial support for this research was provided by NIH grant numbers CA06927 and CA55893 and an appropriation from the Commonwealth of Pennsylvania. In addition, the assistance of Vicki Sayers in preparing the manuscript is appreciated.

### REFERENCES

Brown JM (1979) Evidence for acutely hypoxic cells in mouse tumors and a possible mechanism of reoxygenation. *Br J Radiol* **52**: 650–656

Chaplin DJ, Horsman MR and Aoki DS (1991) Nicotinamide, Fluosol DA and carbogen: a strategy to reoxygenate acutely and chronically hypoxic cells *in vivo*. *Br J Cancer* **63**: 109–113

Chapman JD (1997) Tumor oxygenation. In *Encyclopedia of Cancer*, Vol. III, *Bertino J.* (ed.), pp. 1914–1925. Academic Press: New York

Chapman JD, Dugle DL, Reuvers AP, Meeker BE and Borsa J (1974) Studies on the radiosensitizing effect of oxygen in Chinese hamster cells. *Int J Radiat Biol* **26**: 383–389

Chapman JD, Franko AJ and Sharplin J (1981) A marker for hypoxic cells in tumors with potential clinical applicability. *Br J Cancer* **43**: 546–550

Chapman JD, Franko AJ and Koch CJ (1982) The fraction of hypoxic clonogenic cells in tumor populations. In *Biological Basis and Clinical Implications of Tumor Radioresistance*, Fletcher GH, Nervi C and Withers RH (eds), pp. 61–73. Masson: New York

Chapman JD, McPhee MS, Walz N, Chetner MP, Stobbe CC, Soderlind K, Arnfield M, Meeker BE, Trimble L and Allen PS (1991) NMR spectroscopy and sensitizer-adduct measurements of PDT-induced ischaemia in solid tumours. *J Natl Cancer Inst* **83**: 1650–1659

Chapman JD, Coia LR, Stobbe CC, Englehardt EL, Fenning MC and Schneider RF (1996) Prediction of tumour hypoxia and radioresistance with nuclear medicine markers. *Br J Cancer* **74**: 204–206

Gray LH, Conger AD, Ebert M, Hornsey S and Scott OCA (1953) Concentration of oxygen dissolved in tissues at time of radiation as a factor in radiotherapy. *Br J Radiol* **26**: 628–648

Horsman MR, Brown JM, Hirst VK, Lemmon MJ, Wood PJ, Dunphy EP and Overgaard J (1988) Mechanism of action of selective tumor radiosensitizer nicotinamide. *Int J Radiat Oncol Biol Phys* **15**: 685–690

Horsman MR, Wood PJ, Chaplin DJ, Brown JM and Overgaard J (1990) The potentiation of radiation damage by nicotinamide in the SCC VII tumour *in vivo*. *Radiother Oncol* **18**: 49–57

Hu Q, Kavanagh M-C, Newcombe D and Hill PRP (1995) Detection of hypoxic fractions in murine tumors by comet assay: comparison with other techniques. *Radiat Res* **144**: 266–275

Kavanagh M-C, Sun A, Hu Q and Hill RP (1996) Comparing techniques of measuring tumor hypoxia in different murine tumors: Eppendorf $pO_2$ histograph, [$^3$H]misonidazole binding and paired survival assay. *Radiat Res* **145**: 491–500

Moore RB, Chapman JD, Mokrzanowski AD, McEwan AJ, Arnfield MR and McPhee MS (1992) Non-invasive monitoring of photodynamic therapy with technetium HMPAO scintigraphy. *Br J Cancer* **65**: 491–497

Moore RB, Chapman JD, Mercer JR, Mannon RH, Wiebe LI, McEwan AJ and McPhee MS (1993) Measurement of photodynamic therapy induced hypoxia in Dunning prostatic tumours by Iodine-123-Iodoazomycin arabinoside. *J Nucleic Med* **34**: 405–413

Moulder JE and Rockwell S (1984) Hypoxic fractions of solid tumors. *Int J Radiat Oncol Biol Phys* **10**: 695–712

Rockwell SC, Kallman RF and Fajardo LF (1972) Characteristics of a serially transplanted mouse mammary tumor and its tissue-culture adapted derivative. *J Natl Cancer Inst* **49**: 735–749

Schneider RF, Englehardt EL, Stobbe CC, Fenning MC and Chapman JD (1997) The synthesis and radiolabeling of novel markers of tissue hypoxia of the iodinated azomycin nucleoside class. *J Label Comps Radiopharm* **39**: 541–557

Stone HB, Brown JM, Phillips TL and Sutherland RM (1993) Oxygen in human tumors: correlation between methods of measurement and response to therapy. *Radiat Res* **136**: 422–434

Tannock I and Guttman P (1981) Response of Chinese hamster ovary cells to anticancer drugs under aerobic and hypoxic conditions. *Br J Cancer* **43**: 245–248

Thomlinson RH and Gray LH (1958) The histological structure of some human lung cancers and the possible implications for radiotherapy. *Br J Cancer* **9**: 539–549

Thorndyke C, Meeker BE, Thomas G, Lakey WH, McPhee MS and Chapman JD (1985) The radiation sensitivities of R3327-H and R3327-AT rat prostate adenocarcinomas. *J Urol* **134**: 191–198

Trotter MJ, Chaplin DJ, Durand RE and Olive PL (1989*a*) The use of fluorescent probes to identify regions of transient perfusion in murine tumors. *Int J Radiat Oncol Biol Phys* **16**: 931–934

Trotter MJ, Chaplin DJ and Olive PL (1989*b*) Use of a carbocyanine dye as a marker of functional vasculature in murine tumours. *Br J Cancer* **59**: 706–709

Van Putten LM and Kallman RF (1968) Oxygenation status of a transplantable tumor during fractionated radiation therapy. *J Natl Cancer Inst* **40**: 441–451

Yeh KA, Biade S, Lanciano RM, Brown DQ, Fenning MC, Babb JS, Hanks GE and Chapman DC (1995) Polarographic needle electrode measurements of oxygen in rat prostate carcinomas: accuracy and reproducibility. *Int J Rad Oncol Biol Phys* **33**: 111–118

© Cancer Research Campaign 1998


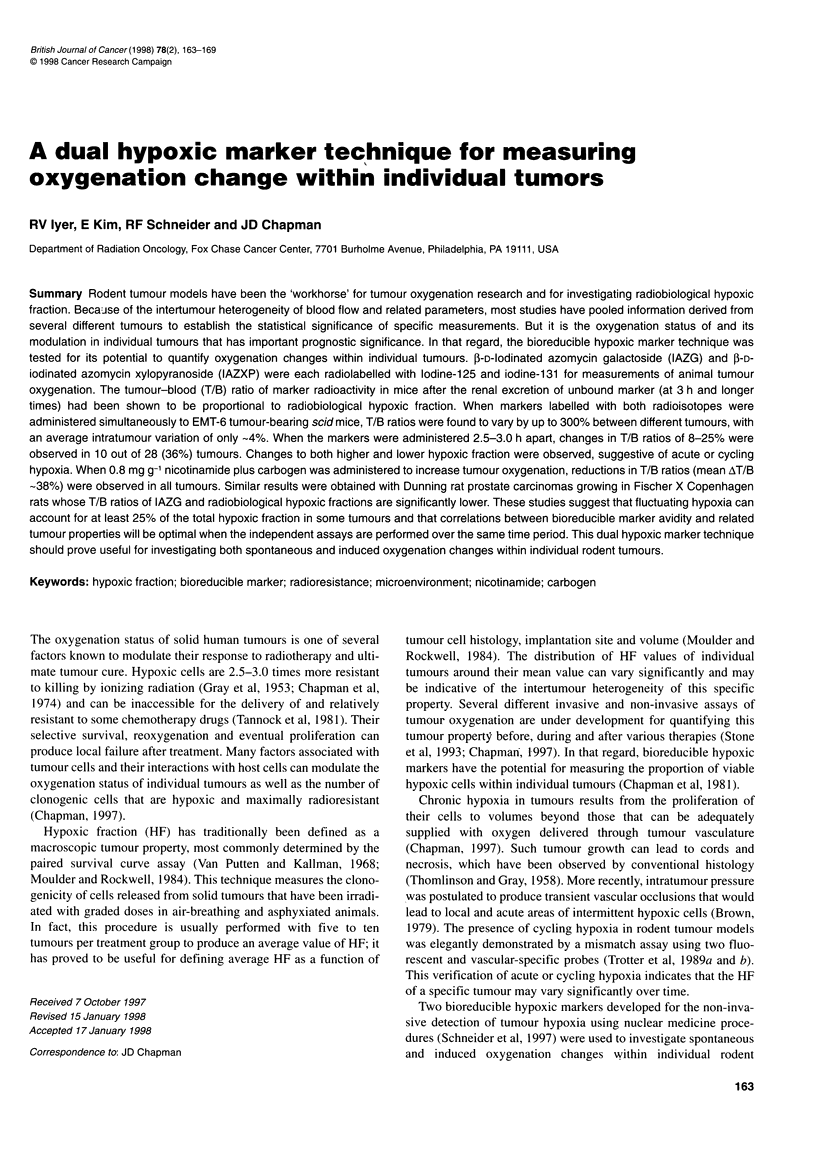

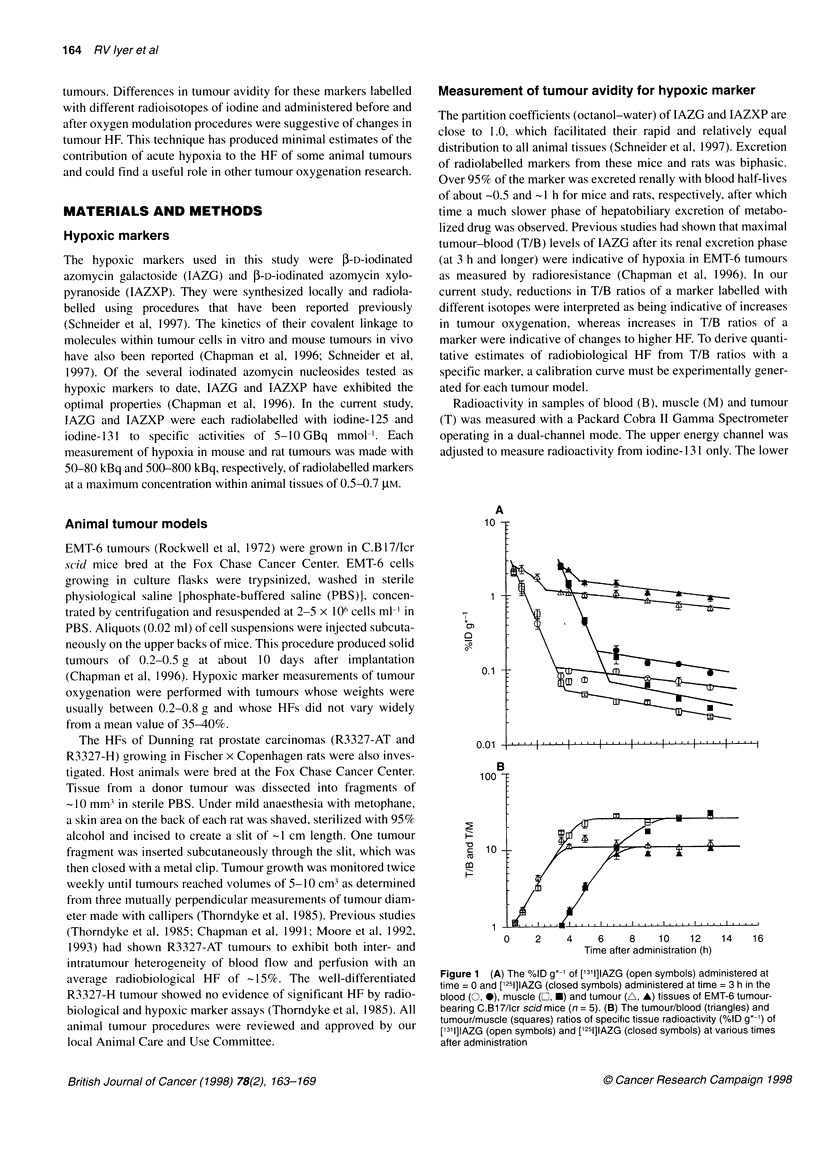

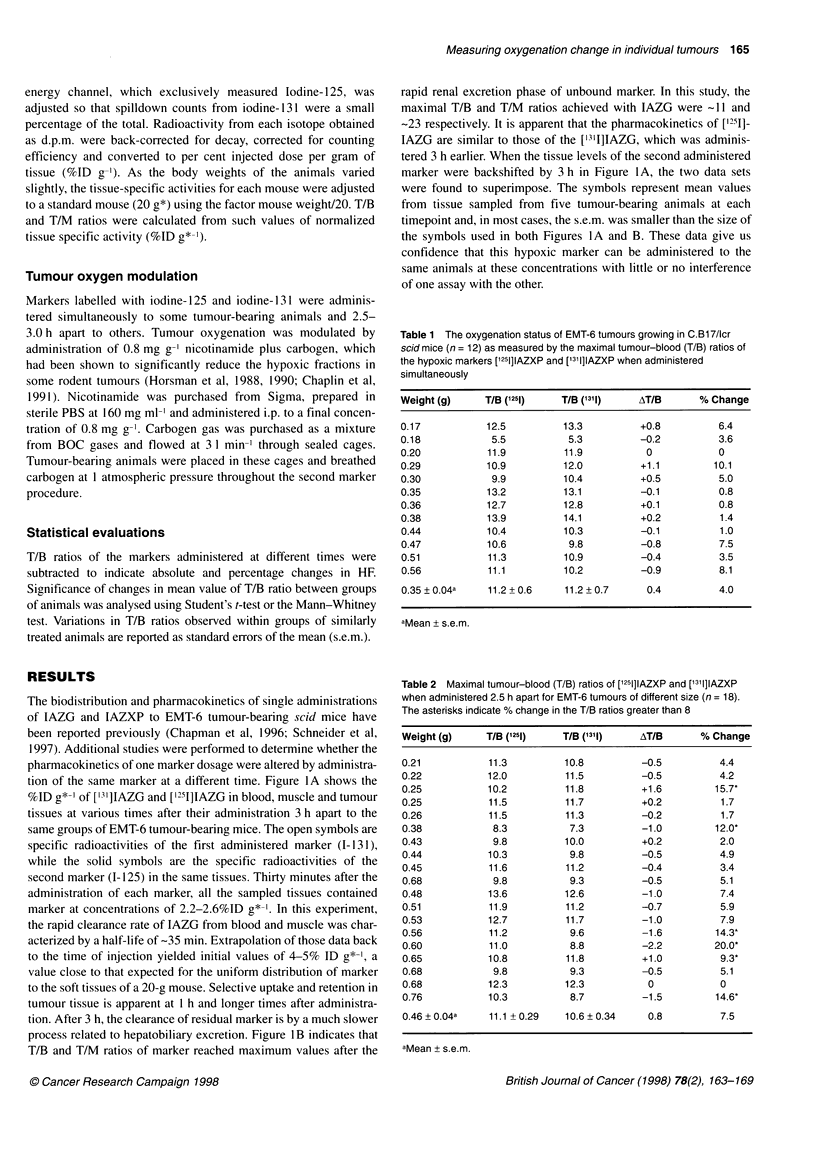

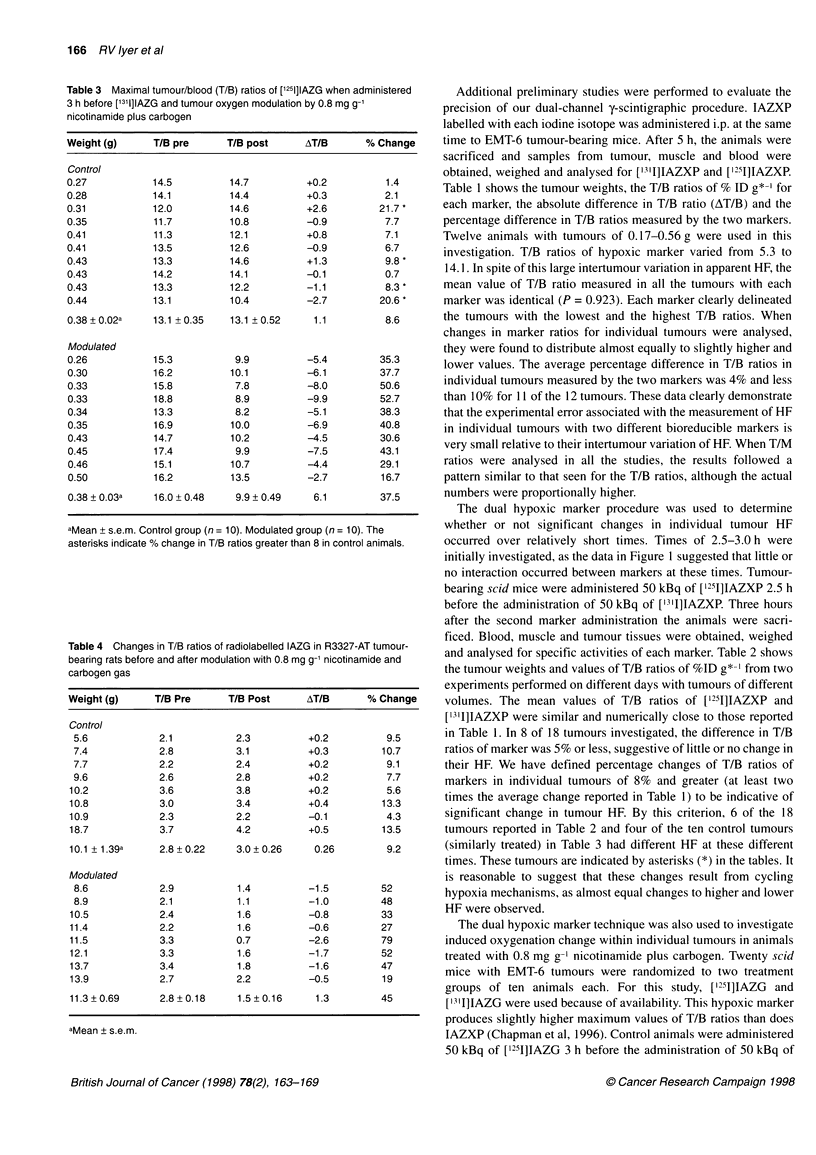

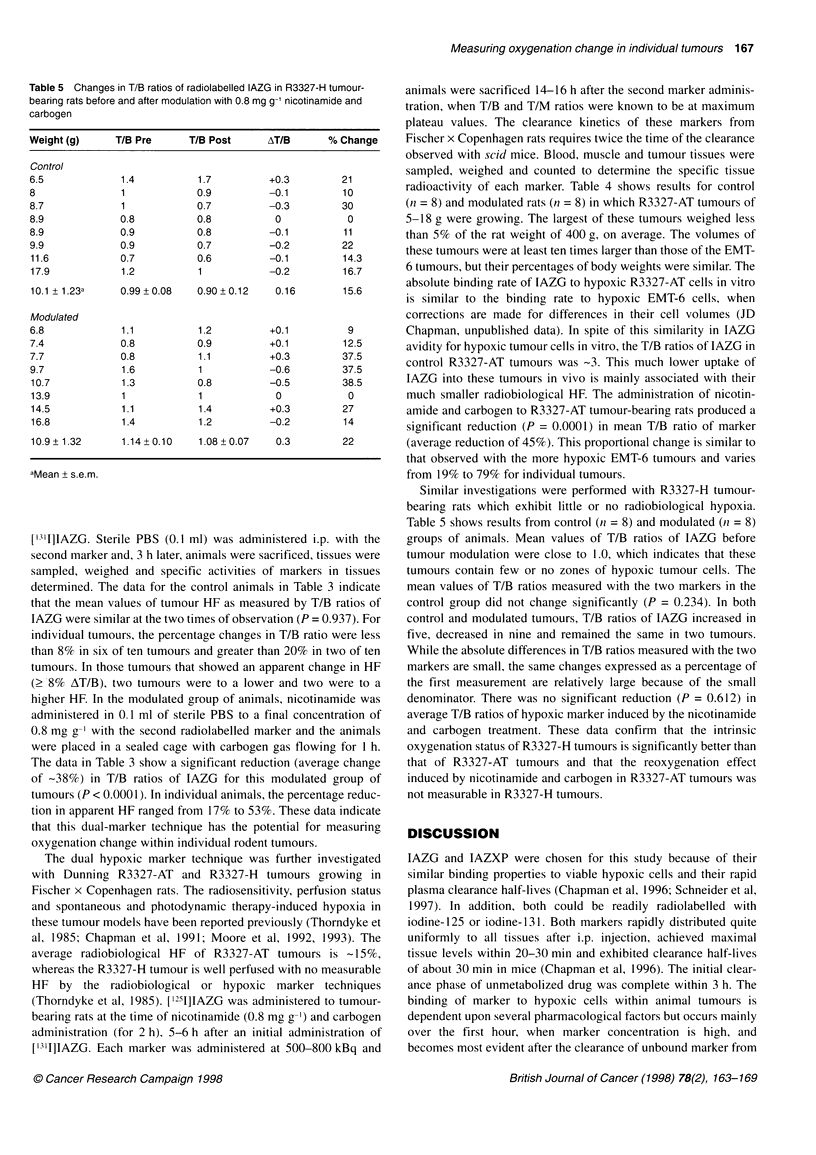

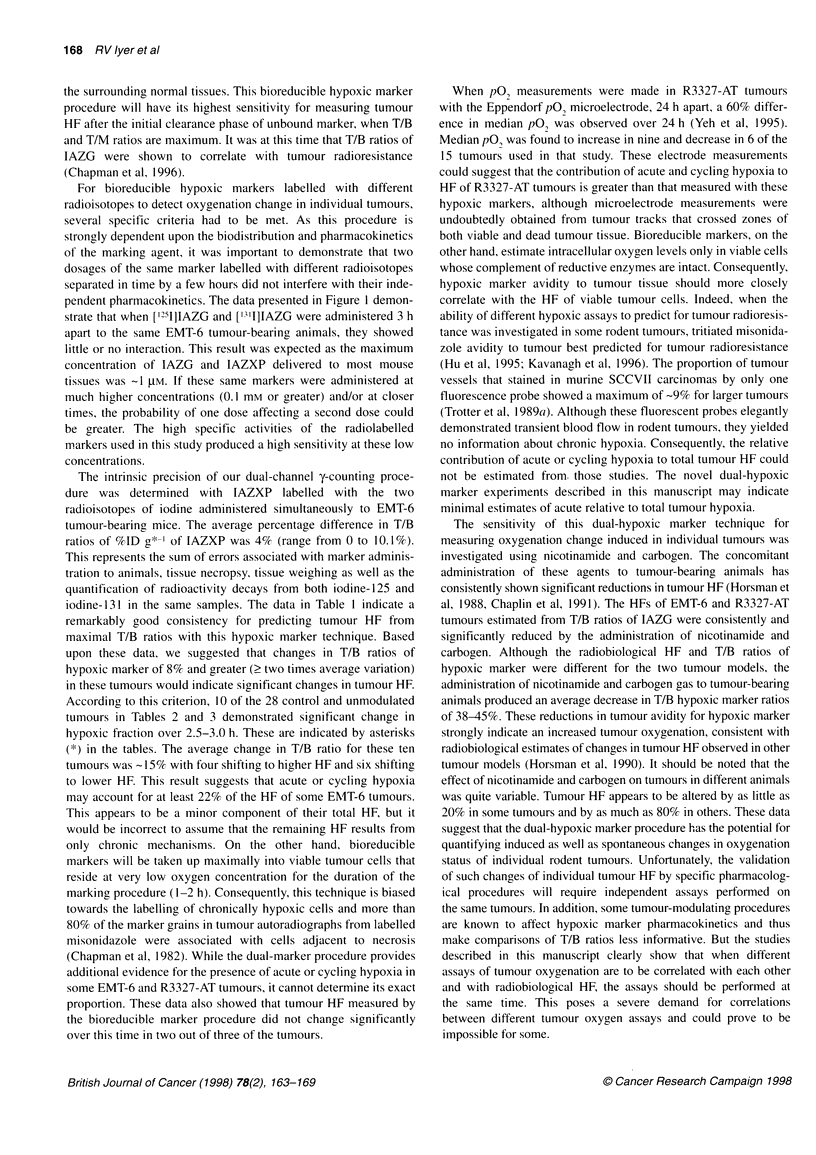

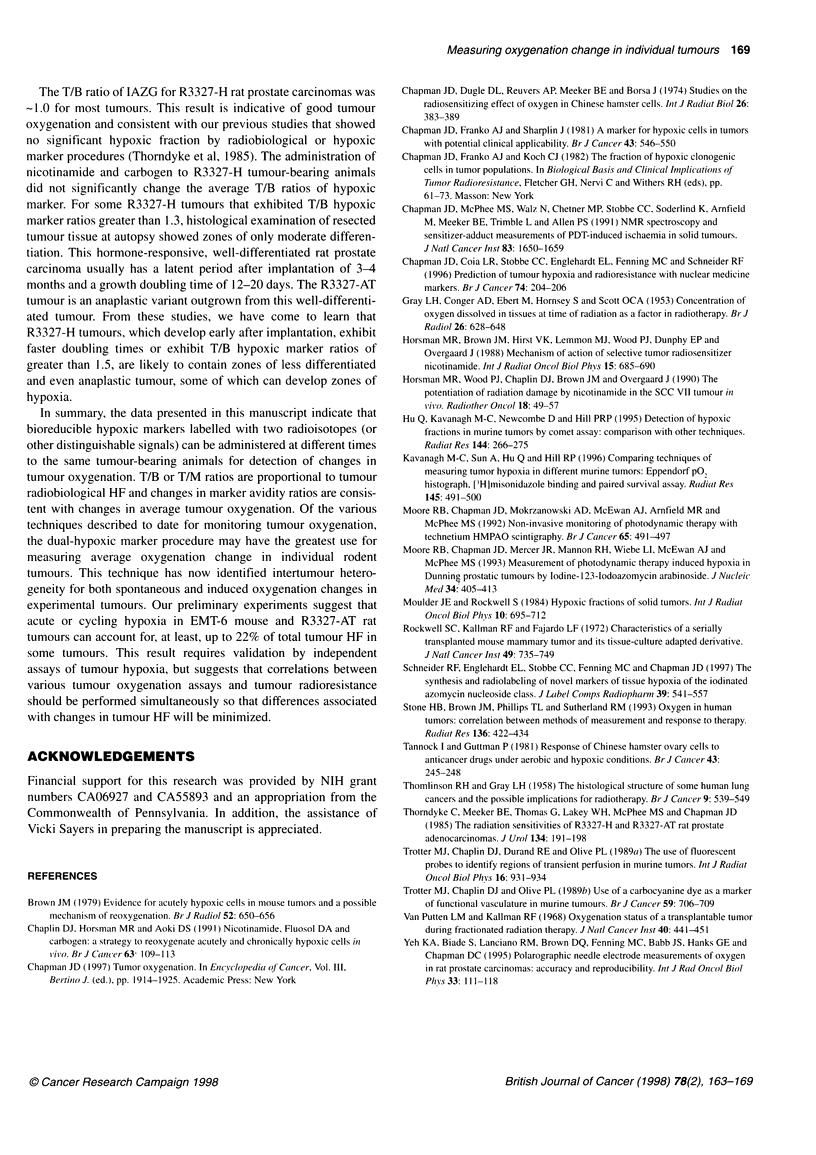


## References

[OCR_00814] Brown J. M. (1979). Evidence for acutely hypoxic cells in mouse tumours, and a possible mechanism of reoxygenation.. Br J Radiol.

[OCR_00818] Chaplin D. J., Horsman M. R., Aoki D. S. (1991). Nicotinamide, Fluosol DA and Carbogen: a strategy to reoxygenate acutely and chronically hypoxic cells in vivo.. Br J Cancer.

[OCR_00827] Chapman J. D., Dugle D. L., Reuvers A. P., Meeker B. E., Borsa J. (1974). Letter: Studies on the radiosensitizing effect of oxygen in Chinese hamster cells.. Int J Radiat Biol Relat Stud Phys Chem Med.

[OCR_00832] Chapman J. D., Franko A. J., Sharplin J. (1981). A marker for hypoxic cells in tumours with potential clinical applicability.. Br J Cancer.

[OCR_00842] Chapman J. D., McPhee M. S., Walz N., Chetner M. P., Stobbe C. C., Soderlind K., Arnfield M., Meeker B. E., Trimble L., Allen P. S. (1991). Nuclear magnetic resonance spectroscopy and sensitizer-adduct measurements of photodynamic therapy-induced ischemia in solid tumors.. J Natl Cancer Inst.

[OCR_00854] GRAY L. H., CONGER A. D., EBERT M., HORNSEY S., SCOTT O. C. (1953). The concentration of oxygen dissolved in tissues at the time of irradiation as a factor in radiotherapy.. Br J Radiol.

[OCR_00859] Horsman M. R., Brown J. M., Hirst V. K., Lemmon M. J., Wood P. J., Dunphy E. P., Overgaard J. (1988). Mechanism of action of the selective tumor radiosensitizer nicotinamide.. Int J Radiat Oncol Biol Phys.

[OCR_00864] Horsman M. R., Wood P. J., Chaplin D. J., Brown J. M., Overgaard J. (1990). The potentiation of radiation damage by nicotinamide in the SCCVII tumour in vivo.. Radiother Oncol.

[OCR_00869] Hu Q., Kavanagh M. C., Newcombe D., Hill R. P. (1995). Detection of hypoxic fractions in murine tumors by comet assay: comparison with other techniques.. Radiat Res.

[OCR_00874] Kavanagh M. C., Sun A., Hu Q., Hill R. P. (1996). Comparing techniques of measuring tumor hypoxia in different murine tumors: Eppendorf pO2 Histograph, [3H]misonidazole binding and paired survival assay.. Radiat Res.

[OCR_00886] Moore R. B., Chapman J. D., Mercer J. R., Mannan R. H., Wiebe L. I., McEwan A. J., McPhee M. S. (1993). Measurement of PDT-induced hypoxia in Dunning prostate tumors by iodine-123-iodoazomycin arabinoside.. J Nucl Med.

[OCR_00881] Moore R. B., Chapman J. D., Mokrzanowski A. D., Arnfield M. R., McPhee M. S., McEwan A. J. (1992). Non-invasive monitoring of photodynamic therapy with 99technetium HMPAO scintigraphy.. Br J Cancer.

[OCR_00892] Moulder J. E., Rockwell S. (1984). Hypoxic fractions of solid tumors: experimental techniques, methods of analysis, and a survey of existing data.. Int J Radiat Oncol Biol Phys.

[OCR_00896] Rockwell S. C., Kallman R. F., Fajardo L. F. (1972). Characteristics of a serially transplanted mouse mammary tumor and its tissue-culture-adapted derivative.. J Natl Cancer Inst.

[OCR_00906] Stone H. B., Brown J. M., Phillips T. L., Sutherland R. M. (1993). Oxygen in human tumors: correlations between methods of measurement and response to therapy. Summary of a workshop held November 19-20, 1992, at the National Cancer Institute, Bethesda, Maryland.. Radiat Res.

[OCR_00916] THOMLINSON R. H., GRAY L. H. (1955). The histological structure of some human lung cancers and the possible implications for radiotherapy.. Br J Cancer.

[OCR_00911] Tannock I., Guttman P. (1981). Response of Chinese hamster ovary cells to anticancer drugs under aerobic and hypoxic conditions.. Br J Cancer.

[OCR_00919] Thorndyke C., Meeker B. E., Thomas G., Lakey W. H., McPhee M. S., Chapman J. D. (1985). The radiation sensitivities of R3327-H and R3327-AT rat prostate adenocarcinomas.. J Urol.

[OCR_00933] Van Putten L. M., Kallman R. F. (1968). Oxygenation status of a transplantable tumor during fractionated radiation therapy.. J Natl Cancer Inst.

[OCR_00937] Yeh K. A., Biade S., Lanciano R. M., Brown D. Q., Fenning M. C., Babb J. S., Hanks G. E., Chapman D. C. (1995). Polarographic needle electrode measurements of oxygen in rat prostate carcinomas: accuracy and reproducibility.. Int J Radiat Oncol Biol Phys.

